# Organ Growth and Intestinal Functions of Preterm Pigs Fed Low and High Protein Formulas With or Without Supplemental Leucine or Hydroxymethylbutyrate as Growth Promoters

**DOI:** 10.3389/fnut.2021.687703

**Published:** 2021-06-04

**Authors:** Randal K. Buddington, Taisiya Yakimkova, Adebowale Adebiyi, Victor V. Chizhikov, Igor Y. Iskusnykh, Karyl K. Buddington

**Affiliations:** ^1^Babies Taking Flight, Memphis, TN, United States; ^2^College of Health Studies, University of Memphis, Memphis, TN, United States; ^3^Department of Physiology, University of Tennessee Health Sciences Center, Memphis, TN, United States; ^4^Department of Anatomy and Neurobiology, University of Tennessee Health Sciences Center, Memphis, TN, United States; ^5^Department of Biological Sciences, University of Memphis, Memphis, TN, United States

**Keywords:** intestine, preterm, pig, development, growth promotor, nutrition, small-for-gestational age

## Abstract

The goal of enteral nutritional support for infants born preterm or small for gestational age (SGA) is to achieve normal growth and development. Yet, this is difficult to achieve because of intestinal immaturity. Our objective was to determine if birth weight, protein intake, and the growth promoters leucine (10 g/L) or calcium-ß-hydroxy-ß-methylbutryate (HMB; 1.1 g/L) would affect trajectories of intestinal growth and functions and weights of other organs. Preterm pigs were delivered at gestational day 105 (91% of term) and fed for 6 or 7 days isocaloric formulas that differed in protein content (50 g or 100 g protein/L), with and without the growth promoters leucine or HMB. For comparative purposes organ weights were measured within 12 h after delivery for six term pigs of low and six of average birth weights. The responses of intestinal growth and total intestinal brush border membrane carbohydrases to protein level and supplemental leucine were of greater magnitude for preterm pigs of lower birth weight. Forskolin stimulated chloride secretion in the proximal small intestine was lower for pigs fed the low protein milk replacers. Capacities of the entire small intestine to transport glucose (mmol/kg-day) were not responsive to protein level, leucine, or HMB, and did not differ between small and large pigs. Relative organ weights of the small and average weight term pigs were similar, but some differed from those of the preterm pigs suggesting preterm birth and the standards of care used for this study altered the trajectories of development for the intestine and other organs. Although leucine is an effective generalized growth promoter that enhances gut development of small preterm pigs, it does not mitigate compromised neurodevelopment. Our findings using preterm pigs as a relevant preclinical model indicate nutrition support strategies can influence development of some gastrointestinal tract characteristics and the growth of other organs.

## Introduction

Being born premature and small-for-gestational-age (SGA) because of intrauterine growth restriction compromises postnatal growth and is the leading cause of infant death and childhood intellectual and developmental disabilities (1, 2). Extrauterine growth retardation (EUGR) caused by inadequate postnatal nutrition further increases the risk of poor outcomes (3–7). The goal of nutrition support for infants born preterm or SGA is to achieve growth and maturation that is similar to *in utero* and thereby improve outcomes (3). Unfortunately, this is often not achieved (8, 9) increasing the risk of death and childhood disability. This can be partly attributed to intestinal immaturity that limits the volumes of enteral nutrition that can be tolerated.

Human fetuses grow rapidly between 22 and 40 weeks of gestation (10). To match the rapid protein accretion of 2 to 2.5 g/kg-d during this period, preterm infants are fed formulas and fortified breast milk that provide 3 to 4 g of dietary protein/kg-d (11–14). Although increasing protein intake of preterm infants beyond 3 g/kg-d can increase weight gains, growth declines when protein intake exceeds 8 g/kg-d, likely by overwhelming the abilities of the immature hepatic and renal systems to handle excessive nitrogenous wastes, potentially causing acidosis, elevated blood urea nitrogen, and hypernatreimic dehydration (15). Similarly, increasing by 50% the protein content of milk replacer fed to SGA term pigs alters serum chemistries and increases mortality, unless supplemented with potassium and phosphorus (16). An alternative strategy that has been evaluated using newborn low birth weight term pigs is activation of the mTOR pathway using leucine and ß-hydroxy-ß-methylbutryate (HMB) to increase protein synthesis (17, 18). The possibility leucine and HMB will enhance intestinal growth and maturation and influence growth of other organs after preterm birth is the focus of the present study.

Preterm pig are used as translational models for human infants (19, 20) and when fed milk replacers with protein content similar to that of mature sow milk realize greater growth than littermates fed milk replacer with double the protein (21). Leucine elicited an even greater growth response to the lower protein milk replacer but did not increase growth when added to the high protein formula that provided 12 g protein per kg-day. Protein synthesis of preterm pigs provided 16 g per kg-d of amino acids is lower compared with term pigs (22). Similar to human infants, fetal and postnatal growth rates are related for pigs (23) and small term pigs grow slower than normal birth weight littermates (24). A novel finding from the previous study was the growth response to the low protein milk replacer with leucine was of greater magnitude for smaller preterm pigs. What has not been understood is how postnatal growth of the intestine and other organs after preterm birth is influenced by birth weight, dietary protein content, and growth promoters despite the realization of the potential lifelong consequences of delayed organ growth, particularly for the brain (25) and kidneys (26). The results presented herein used organs harvested from the same preterm pigs to investigate the responses of the immature intestine and growth of other organs to milk replacers with different protein content with and without the growth promoters leucine and HMB. Newborn term pigs of average body weight and SGA were used for comparisons of organ weights.

## Materials and Methods

All phases of the research using animals was approved by the Institutional Animal Care and Use Committee of the University of Memphis (approval # 0674). We recorded weights of the small intestine and other selected organs (liver, lungs, heart, kidneys, spleen, pancreas, stomach, total brain, and cerebellum) and evaluated small intestine functions of a total of 105 preterm pigs from 11 litters that were delivered 10 days prior to term (day 105 of 115-day term) and had been used to evaluate body weight gain in response to being fed milk replacers with low and high protein levels (50 and 100 g/L), with and without supplemental leucine (10 g/L) or HMB (1.1 g/L) to stimulate mTORC1 (21). Each litter was distributed to three sizes (small, intermediate, and large) and pigs in each size group were randomly distributed to one of three treatment groups so that each treatment had comparable size distributions to account for the influence of birthweight on postnatal growth (24). Briefly, parenteral nutrition was provided for 24 h before starting full enteral nutrition with bolus feeding (15 ml/kg every 3 h) of milk replacers. The milk replacers were fed for 6 or 7 days with volumes adjusted daily based on weight gains.

Pigs in the initial five litters were distributed to two low protein milk replacers with either alanine (LP-Ala) or leucine (LP-Leu) and the high protein milk replacer (HP), with sample sizes at necropsy of 13, 18, and 16, respectively. The LP-Leu and HP milk replacers had comparable leucine content but were not isonitrogenous. Alanine was used as a control amino acid as it is not associated with any known signaling pathway that elicits growth responses and was included at 6.8 g/L to be equimolar with the added leucine. The two LP milk replacers were supplemented with medium chain triglyceride oil to be isocaloric with the HP milk replacer. In response to the lower growth elicited by the HP milk replacer compared with the two LP milk replacers a subsequent six litters were harvested to determine if supplementing the high protein milk replacer with leucine (HP-Leu) or HMB (HP-HMB) would improve growth compared to a control HP with alanine (HP-Ala), with sample sizes at necropsy of 20, 15, and 23. Ca-acetate was added to the HP-Ala and HP-Leu milk replacers to balance the additional Ca associated with the HMB and maintain a Ca:P ratio below 2. Lactose was the sole source of carbohydrate in the milk replacers to reduce the risk of necrotizing enterocolitis (27). The pigs were euthanized after the period of enteral feeding and the small intestine and the stomach, liver, heart, lungs, kidneys, total brain and isolated cerebellum, pancreas, and spleen were collected.

An additional 12 newborn term pigs were obtained within 12 h after delivery for comparison of organ weights, with 6 of the pigs considered as small for gestational age (SGA; <1,000 g) and the remaining 6 pigs were littermates of average birthweight (AGA; >1,200 g). The term pigs were used to determine if organ weights of preterm pigs at near term equivalent post conception age (112 days) were more similar to those of term SGA or to AGA pigs (115 days); small intestine functions were not evaluated for the term pigs.

### Assessment of Small Intestine Size and Functions

The small intestines of the preterm pigs from the pyloric sphincter to the ileocolonic junction was removed. After severing of the associated mesenteries, the total length was recorded on a horizontal surface, and the small intestine was divided into three regions of equal lengths that were designated as proximal, mid, and distal. Each segment of small intestine was flushed using cold Ringers, excess fluid was gently squeezed out while the segment was held vertical, before weight was recorded. A segment from the middle of each region was flash frozen in liquid nitrogen for later analysis of carbohydrases associated with the brush border membrane (BBM). An adjacent segment in each region was used for *in vitro* measurements of glucose uptake. An additional segment from the proximal region was used for Ussing chamber measurements of forskolin-induced chloride secretion. The colon was examined for NEC lesions or other pathologies but was not weighed. Weights were recorded for other harvested organs.

Carbohydrase activities were based on the release of glucose from lactose, maltose, and maltodextrin (average degree of polymerization of 5) by brush border membrane vesicles prepared from the frozen segments from the three small intestine regions (28). Carbohydrase activities in each region were integrated with regional weights to estimate regional capacities and these were summed to estimate the total intestinal activity of each carbohydrase.

Rates of glucose uptake were measured as before (29) using intact sleeves of everted intestine exposed to the saturating concentration of 50 mM to determine maximum rates of uptake. Rates of glucose uptake (nmol D-glucose absorbed per min) represent carrier-mediated uptake, presumably via the sodium-dependent transporter, SGLT-1. Rates of uptake per cm by the three regions were integrated with intestinal length to estimate total capacities of the entire small intestine to transport glucose.

Chloride secretion by the proximal small intestine was measured using an Ussing chamber system (Physiological Instruments) after the serosa and muscularis were removed and with mammalian Ringers in both chambers. After a stable baseline short circuit current was established, forskolin was added (10 μM final concentration) to stimulate chloride secretion. The change in short circuit current (ΔIsc) was used as an indicator of stimulated chloride secretion.

### Statistical Analysis

Values in tables are means and standard deviations. The data were analyzed by ANCOVA with litter and body weight included as potential confounders of responses to treatment. Unpaired *t*-tests were used for selected comparisons. *P* < 0.05 was accepted as the critical level of significance.

## Results

Rates of body weight gain by the preterm pigs over the 6 or 7 days of enteral nutrition have been reported (21). Briefly, body weights at birth and necropsy and rates of weight gain were similar for the four groups of preterm pigs fed the high protein formulas (HP, HP-Ala, HP-Leu, HP-HMB; Table 1). When all pigs were included, at birth the preterm pigs fed the HP diets tended to be slightly larger than the LP preterm pigs (1008 g ± 299 vs. 895 ± 264; *P* = 0.07). Due to significantly greater rates of daily weight gain for the preterm pigs fed the LP milk replacer [2.44%/day ± 0.73 vs. 3.40 ± 0.55; *P* < 0.0001; (21)], the difference in body weights was diminished at necropsy (1326 g ± 419 vs. 1231 ± 372; *P* = 0.28). The enhanced daily weight gain was more pronounced for preterm pigs fed the LP milk replacer with leucine. The HP pigs in the initial five litters did not differ from the HP-Ala pigs in the subsequent six litters for growth or any of the measured organs. These two groups were pooled for all further comparisons and labeled hereafter as HP-Controls. The number of preterm pigs that were <1,000 g at necropsy (*n* = 24) represented about 23% of the total number of pigs studied. The supplier informed us only about 10% of newborn term pigs delivered at the facility are <1,000 g and these are considered as “small.” The higher percentage of preterm pigs that were <1,000 g at near term equivalent age is consistent with the lower growth rate of preterm pigs and preterm infants, resulting in being smaller at term equivalent ages.

**Table 1:** Body weights (g) at delivery and necropsy and daily percent weight gains and small intestine lengths (cm) and weights (g) of preterm pigs fed the milk replacers with high (HP) and low (LP) protein with leucine (Leu) or calcium-ß-hydroxy-ß-methylbutryate (HMB) as growth promoters or alanine (Ala; control).

	**LP-Ala**	**LP-Leu**	**HP-Control**	**HP-Leu**	**HP-HMB**
Birth weight	933 ± 300	868 ± 240	960 ± 342	1086 ± 253	1031 ± 214
Death weight	1255 ± 414	1214 ± 349	1263 ± 467	1437 ± 382	1347 ± 300
% daily weight gain	3.10 ± 0.58^b^	3.62 ± 0.43^b^	2.44 ± 0.86^a^	2.42 ± 0.49^a^	2.49 ± 0.65^a^
Small intestine length	396 ± 86 (311 ± 42)	391 ± 61 (339 ± 70)	403 ± 80 (351 ± 95)	437 ± 57 (318 ± 68)	426 ± 57 (325 ± 54)
Small Intestine weight	50.0 ± 17.8 (40.7 ± 4.7)	49.9 ± 14.1 (42.1 ± 6.4)	47.5 ± 17.0 (39.6 ± 5.6)	55.7 ± 16.1 (38.7 ± 5.2)	50.8 ± 13.7 (37.6 ± 5.0)

*Values in parentheses are normalized small intestine lengths (cm/kg) and weights (g/kg). Values with different letter superscripts are significantly different*.

**Figure 1:**
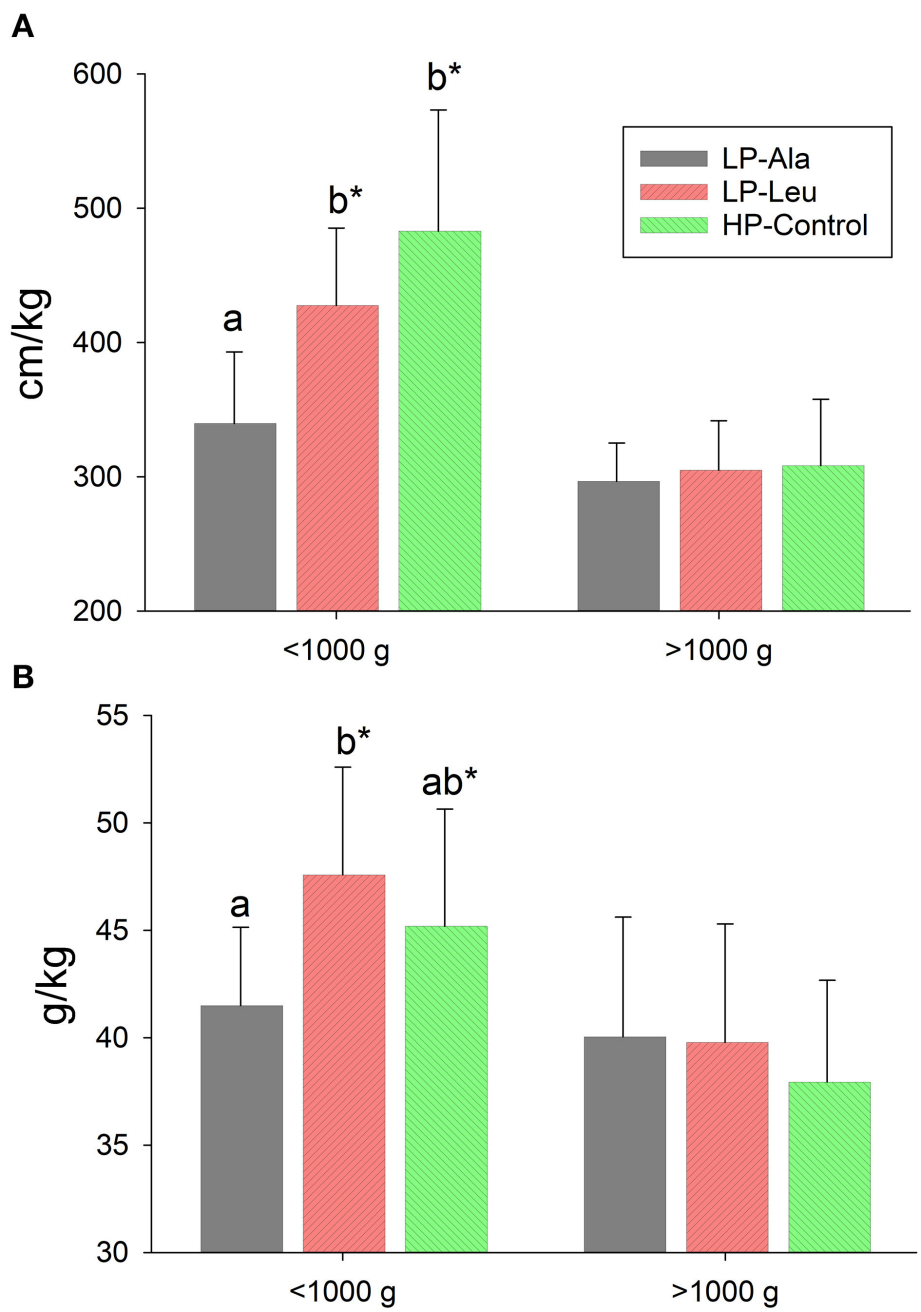
Small intestine lengths **(A)** and weights **(B)** normalized to body weight for preterm pigs less than and greater than 1,000 g at necropsy that had been fed milk replacers with low protein with either alanine (LP-Ala) or leucine (LP-Leu) or high protein (HP-Control). Bars with different letters are significantly different. Asterisks denote values for pigs <1,000 g differ from those >1,000 that were fed the same milk replacer.

The potential confounding factor of necrotizing enterocolitis on intestine structure and functions (30) was not observed in this study using milk replacers with lactose as the sole source of carbohydrate. When all pigs in the low and high diet groups were pooled small intestine length (cm) tended to be longer for pigs fed the HP diets (417 ± 71 vs. 393 ± 71; *P* = 0.07) but not when normalized to body weight (337 ± 82 vs. 328 ± 61; *P* = 0.69). There were no differences between the individual treatment groups when all pigs were included for comparisons. In light of the greater growth rate of the smaller LP pigs, we compared small intestine lengths normalized to body weight for LP and HP pigs that were less than or greater than 1,000 g at necropsy. This comparison revealed the small intestine was relatively longer (cm/kg) for smaller compared with larger LP pigs (388 ± 70 vs. 302 ± 33; *P* < 0.001) with a similar size related difference for HP pigs (456 ± 83 vs. 309 ± 50; *P* < 0.001). Supplementing the LP milk replacer with leucine rather than alanine resulted in relatively longer intestine for comparisons of pigs <1,000 g (Figure 1), with resulting lengths not different from those of the similar sized HP-Control pigs. The response to leucine and higher protein was absent for the larger pigs. Small intestine lengths (absolute and normalized) did not differ among the HP-Control, HP-Leu and HP-HMB pigs.

Absolute weight of the small intestine did not differ between HP and LP groups (50.6 g ± 16.2 vs. 49.9 ± 15.5; *P* = 0.85), whereas relative intestinal mass (g/kg) was greater for LP compared with HP preterm pigs (38.9 g/kg ± 5.4 vs. 41.5 ± 5.7; P = 0.03). The smaller pigs <1,000 g that were fed the LP and HP milk replacers had relatively heavier intestines than the larger pigs in each group (p's = 0.03 and 0.01, respectively). The small intestines of LP-Leu and HP-Control pigs were relatively heavier than those of LP-Ala pigs (Figure 1). Supplementing the HP formula with leucine or HMP did not increase intestinal weight (absolute or relative to body weight) compared with the HP-Control.

Comparisons of relative weights for the other harvested organs (Table 2) revealed pigs fed the LP milk replacers had smaller kidneys (7.2 g/kg ± 0.8 vs. 8.56 ± 1.6; *P* < 0.001) and spleens (3.8 g/kg ± 1.3 vs. 4.41 ± 1.4; *P* = 0.05) than pigs fed the HP milk replacers. The smaller size of kidneys of LP pigs was pronounced for body weights >1,000 g (7.1 ± 0.8 vs. 8.8 ± 1.6; *P* < 0.001) whereas LP and HP pigs <1,000 g had kidneys of similar size. The livers of LP-Leu pigs were relatively smaller than those of LP-Ala pigs (29.8 g/kg ± 5.7 vs. 33.8 g/kg ± 4.4; *P* < 0.05). Relative weights of the other organs were similar among the HP-Control, HP-Ala, and HP-Leu pigs.

**Table 2:** Weight of other organs (g) harvested during the necropsies of preterm pigs fed the milk replacers with high (HP) and low (LP) protein with leucine (Leu) or calcium-ß-hydroxy-ß-methylbutryate (HMB) as growth promoters or alanine (Ala; control).

**Organ**	**LP-Ala**	**LP-Leu**	**HP-Control**	**HP-Leu**	**HP-HMB**
Stomach	8.02 ± 2.78 (6.40 ± 0.58)	8.1 ± 2.2 (6.6 ± 0.9)	8.1 ± 2.8 (6.5 ± 0.9)	9.1 ± 1.9 (6.4 ± 0.8)	8.2 ± 1.5 (6.1 ± 0.7)
Liver	43.5 ± 18.5 (33.8 ± 4.5)^a^	37.1 ± 13.7 (29.8 ± 5.7)^b^	42.1 ± 16.7 (32.2 ± 4.8)^b^	44.4 ± 13.3 (31.0 ± 4.1)^b^	42.0 ± 12.7 (30.8 ± 4.0)^b^
Heart	9.2 ± 3.3 (7.3 ± 0.7)	8.7 ± 2.2 (7.3 ± 0.9)	9.2 ± 3.5 (7.2 ± 0.7)	10.1 ± 3.4 (6.9 ± 0.9)	9.4 ± 2.1 (7.0 ± 0.7)
Lungs	24.4 ± 6.8 (20.1 ± 3.8)	23.7 ± 8.2 (19.6 ± 4.1)	25.0 ± 8.7 (20.2 ± 3.4)	29.5 ± 9.9 (20.7 ± 4.8)	27.2 ± 6.4 (20.4 ± 3.4)
Kidneys	9.3 ± 3.4^a^ (7.3 ± 0.8)^a^	8.5 ± 2.6^a^ (7.1 ± 0.9)^a^	11.1 ± 5.2^b^ (8.6 ± 1.7)^b^	12.1 ± 3.8^b^ (8.5 ± 1.8)^b^	11.8 ± 3.6^b^ (8.7 ± 1.3)^b^
Total brain	30.3 ± 2.5 (27.0 ± 9.5)	30.4 ± 2.1 (27.3 ± 8.7)	29.9 ± 2.9 (27.3 ± 11.6)	30.3 ± 2.1 (22.4 ± 5.7)	30.3 ± 1.4 (23.6 ± 5.6)
Cerebellum	3.37 ± 0.83 (2.99 ± 1.19)	3.26 ± 0.70 (2.86 ± 0.80)	3.21 ± 0.69 (2.89 ± 1.18)	3.39 ± 0.55 (2.50 ± 0.67)	3.24 ± 0.47 (2.50 ± 0.59)
Pancreas	3.59 ± 1.72 (2.71 ± 0.64)	2.89 ± 1.38 (2.28 ± 0.81)	3.13 ± 1.35 (2.45 ± 0.47)	3.58 ± 1.15 (2.49 ± 0.61)	3.27 ± 1.05 (2.34 ± 0.46)
Spleen	5.74 ± 3.49 (4.22 ± 1.60)	4.48 ± 2.17 (3.53 ± 1.10)	5.68 ± 3.11 (4.28 ± 1.61)	6.70 ± 2.54 (4.60 ± 1.13)	6.12 ± 2.29 (4.46 ± 1.02)

*Values in parentheses are organ weights normalized to body weights (g/kg). Values with different letter superscripts are significantly different*.

Pooled data for organ weights and small intestinal lengths of the preterm pigs fed the high and low protein milk replacers with and without Leu and HMB were plotted as log × log plots with final body weight (Table 3). The slopes for the liver, kidneys, pancreas, and especially the spleen exceeded a value of 1.0, indicating these tissues represented a larger proportion of body mass in the larger preterm pigs. Heart weight remained a constant proportion of body mass across all preterm pigs. The stomach, small intestine, and lungs grew at a rate slightly more than the predicted exponent of body weight^0.75^ based on the weight specific increase in metabolic rate. The weights of the entire brain and isolated cerebellum varied little over the wide range of body weights. Since the preterm pigs were studied at the same post conception age, the similar brain and cerebellar weights indicate the trajectory of brain growth is dependent on gestation age and independent of gains in body weight. Only the smallest pigs (<1,000 g) had brains that were smaller (27.4 g vs. 30.7 g for pigs >1,000 g; *P* < 0.001), with smaller cerebella (2.9 g vs. 3.5 g; *P* < 0.05).

**Table 3:** Slopes, correlation coefficients, and *P*-values for log × log plots of body weights vs. organ measurements recorded at necropsy for all preterm pigs fed the high and low protein milk replacers.

**Organ**	**Slope and SE**	***R*^**2**^**	***P***
Stomach weight	0.85 ± 0.03	0.88	<0.0001
Small intestine length	0.46 ± 0.03	0.74	<0.0001
Small intestine weight	0.87 ± 0.04	0.84	<0.0001
Liver weight	1.18 ± 0.04	0.88	<0.0001
Heart weight	0.99 ± 0.03	0.91	<0.0001
Lung weight	0.86 ± 0.05	0.76	<0.0001
Kidney weight	1.11 ± 0.05	0.81	<0.0001
Total brain weight	0.13 ± 0.02	0.32	<0.0001
Cerebellum weight	0.20 ± 0.05	0.13	<0.0001
Pancreas weight	1.14 ± 0.06	0.76	<0.0001
Spleen weight	1.49 ± 0.08	0.75	<0.0001

The SGA term pigs weighed <50% of the AGA term pigs (Table 4; *P* < 0.001; range 695 g to 914 g vs. 1,240 g to 1,998 g) and were even smaller than the average for the preterm pigs at the time of necropsy (*P* < 0.01). The AGA term pigs had significantly larger intestines and other organs than the SGA pigs, but the differences were not apparent when organ weights were normalized to body mass. The AGA and SGA term pigs had proportionally smaller intestines, livers, and spleens, but larger hearts and kidneys compared to the preterm pigs that had been fed for 6 or 7 days (*P*'s < 0.05). The intact brains and isolated cerebella of the term AGA were heavier than those of the preterm pigs (*P*'s < 0.01), whereas the total brain weights of term SGA pigs were similar to those of the preterm pigs and the cerebella were smaller (*P* < 0.05).

**Table 4:** Body (kg) and organ (g) weights of newborn term pigs of average and low birth weights.

	**AGA**	**SGA**
Birth weight (kg)	1.64 ± 0.11^a^	0.74 ± 0.031^b^
Stomach Mass (g)	6.59 ± 1.72 (4.00 ± 0.93)	4.62 ± 0.83 (6.02 ± 0.39)
Sm Int Wt (g)	54.20 ± 9.64^a^ (31.67 ± 2.20)	23.4 ± 5.3^b^ (32.2 ± 7.8)
Liver (g)	44.39 ± 3.91^a^ (23.49 ± 2.10)	17.19 ± 1.05^b^ (27.77 ± 3.57)
Heart (g)	15.52 ± 0.89^a^ (9.71 ± 1.02)	6.67 ± 0.45^b^ (9.09 ± 0.75)
Lungs (g)	29.24 ± 1.88^a^ (18.22 ± 1.73)	16.36 ± 1.45^b^ (22.36 ± 2.29)
Kidneys (g)	15.87 ± 2.19^a^ (9.61 ± 1.18)	7.08 ± 0.90^b^ (9.96 ± 1.75)
Spleen (g)	2.71 ± 0.35^a^ (1.65 ± 0.16)	0.98 ± 0.09^b^ (1.35 ± 0.16)
Total brain (g)	33.86 ± 0.51a^a^ (21.18 ± 1.63)^a^	29.95 ± 0.30^b^ (40.83 ± 1.97)^b^
Cerebellum (g)	3.59 ± 0.16^a^ (2.26 ± 0.22)^a^	2.85 ± 0.17^b^ (3.89 ± 0.32)^b^

*Values in parentheses are organ weights normalized to body weights (g/kg). Values with different letter superscripts are significantly different*.

### Intestinal Functions

**Figure 2:**
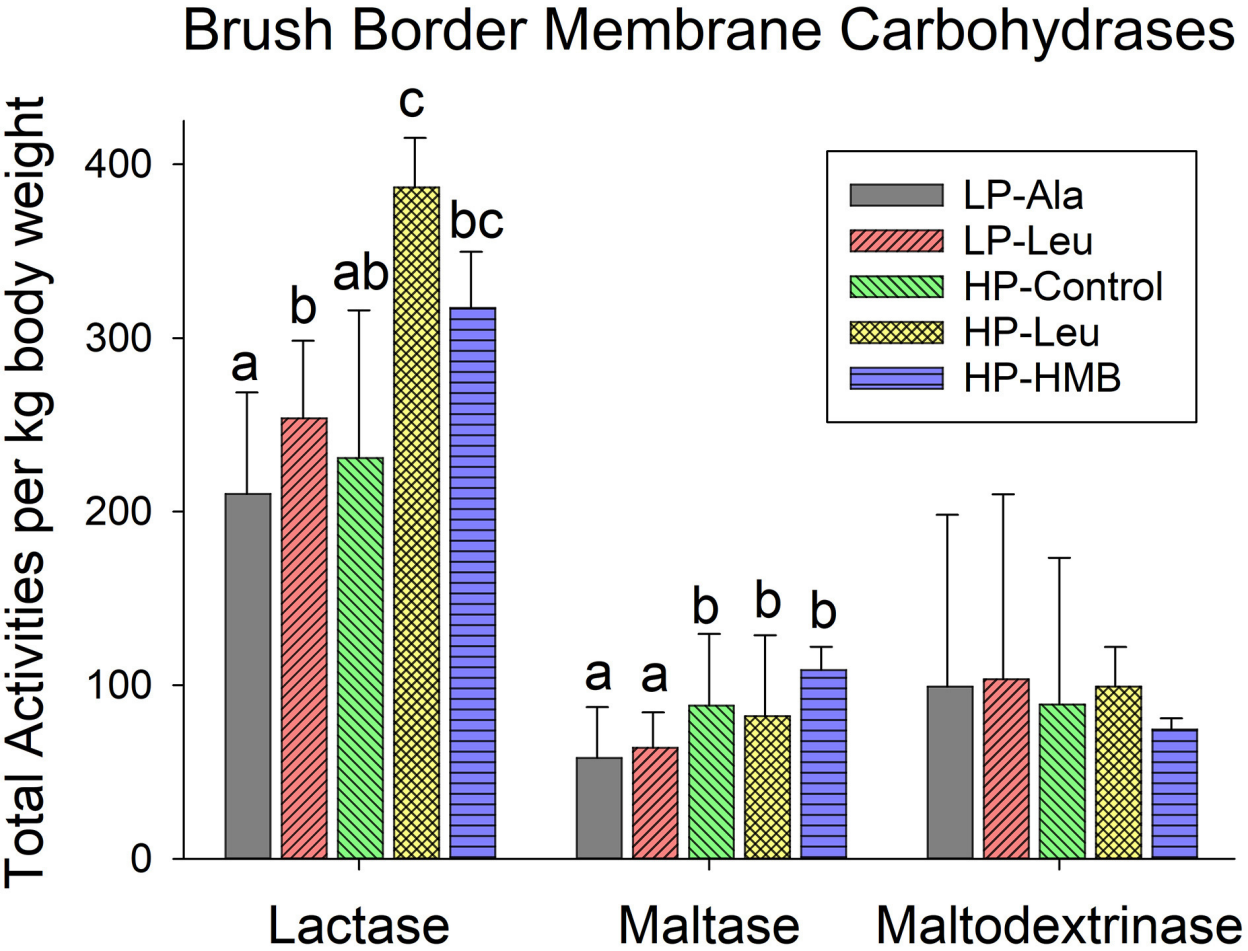
Total small intestine brush border membrane carbohydrase activities measured for preterm pigs fed the milk replacers with low (LP) or high (HP) protein with leucine (Leu) or calcium-ß-hydroxy-ß-methylbutryate (HMB) as growth promoters or alanine (Ala; control). Bars for each enzyme with different letter superscripts are significantly different.

**Figure 3:**
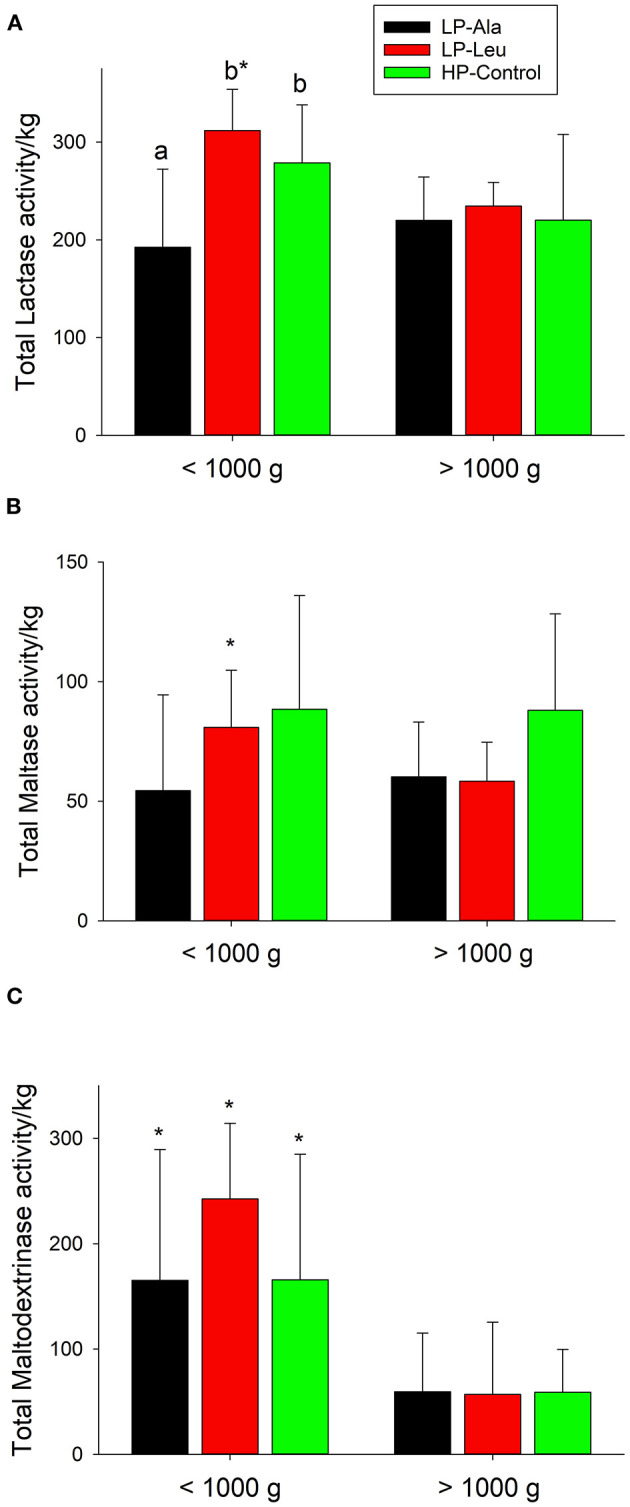
Total small intestine brush border membrane activities normalized to body weights for lactase **(A)**, maltase **(B)**, and maltodextrinase **(C)** and for preterm pigs weighing less than and greater than 1,000 g that were fed the milk replacers with low (LP) or high (HP) protein with leucine (Leu) or calcium-ß-hydroxy-ß-methylbutryate (HMB) as growth promoters or alanine (Ala; control). Bars with different letters are significantly different. Asterisks denote values for pigs <1,000 g differ from those >1,000 that were fed the same milk replacer.

To account for variation in sizes of the preterm pigs the comparisons of activities of brush border membrane carbohydrases and rates of glucose were made using total small intestinal capacities that were normalized to body weights. Lactase was the dominant brush border membrane carbohydrase, with the highest activities measured in pigs fed the HP milk replacers supplemented with leucine and HMB (Figure 2). Adding leucine to the LP milk replacer also increased lactase activities relative to adding alanine. Separating the preterm pigs fed the LP milk replacer with leucine into groups of smaller and larger animals (<1,000 g and > 1,000 g and averaging 754 g ± 234 and 1482 ± 262, respectively) revealed a size related response. Specifically, the increased lactase activities in response to leucine was restricted to the smaller pigs with no response for the larger pigs (Figure 3A). The HP-Control milk replacer tended to elicit higher lactase activities in the smaller compared to the larger pigs (P = 0.07). There were insufficient numbers of small pigs fed the HP milk replacers with leucine and HMB to make similar comparisons.

Preterm pigs fed the LP milk replacers with alanine and leucine had lower maltase activities compared to those fed the HP-Control milk replacers (Figure 2; *P*'s = 0.02 and 0.04, respectively). Again, separating the LP pigs into small and large groups revealed smaller pigs responded to leucine with higher maltase activities compared with alanine (Figure 3B). Maltase activities did not differ for larger pigs fed the LP milk replacers with alanine and leucine with both lower than larger HP-Control pigs.

The apparent lack of differences for maltodextrinase among the five different diet groups (Figure 2) obscured how smaller pigs fed the two LP and the HP-Control milk replacers had higher activities compared with larger pigs fed the same milk replacers (Figure 3C). Moreover, supplementing the LP milk replacer with leucine resulted in even higher activities that exceeded those of LP-alanine and HP-Control pigs.

The capacities of the entire small intestine to actively transport glucose did not differ among preterm pigs fed the two LP and the three HP milk replacers. Nor did the capacities relative to body weight of the smaller preterm pigs differ from those of larger pigs fed the same milk replacer.

**Figure 4:**
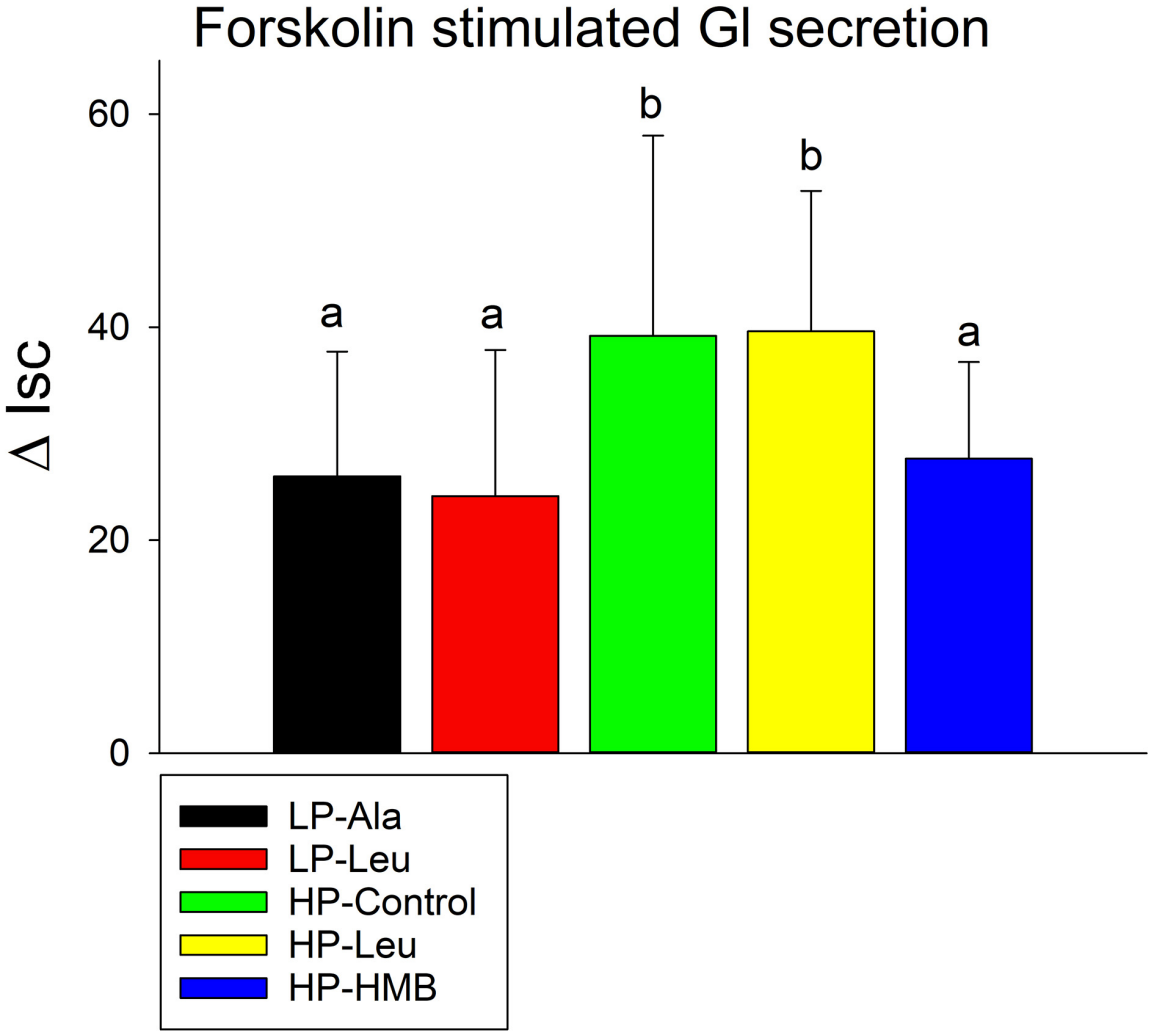
Forskolin stimulated increases in short circuit currents (Isc) were measured using proximal small intestine tissues from preterm pigs that were fed the milk replacers with low (LP) or high (HP) protein with leucine (Leu) or calcium-ß-hydroxy-ß-methylbutryate (HMB) as growth promoters or alanine (Ala; control). Bars with different letters are significantly different.

Forskolin stimulated chloride secretion (Figure 4) did not differ for the preterm pigs fed the LP milk replacers with alanine and leucine. Both had lower short circuit currents responses compared to the HP-Control and HP-Leu pigs that were also similar. Supplementing the HP milk replacer with HMB decreased the response to forskolin. The responses of small pigs to forskolin did not differ from those of larger pigs.

## Discussion

The immaturity of the underdeveloped gastrointestinal tract of preterm pigs and preterm infants limits the dietary loads that can be tolerated and has been implicated in necrotizing enterocolitis (30). Hence, nutritional interventions that accelerate gastrointestinal growth and functional maturation after preterm birth are of obvious interest, particularly after prolonged reliance on total parenteral nutrition compromises gastrointestinal development (31). Although breast milk is the preferred source of nutrition for preterm and term infants, it is not universally available and currently available formulas may not elicit normal development and maturation and increase the risk of necrotizing enterocolitis. Needed advances in nutrition support have been constrained by ethical considerations limit how preterm infants can be used to explore responses to diet composition. The relatively recent recognition of preterm pigs as a relevant and translational model for preterm infants (19, 20) is accelerating the understanding of the interactions between diet and the trajectory of intestinal growth and maturation after preterm birth and the implications in health and disease. The present study corroborates the disconnects that exist between growth of the intestine and development of some functions (32). An important consideration is how the influence of nutrition differs between preterm and term pigs for some intestinal characteristics (31), limiting the extrapolation of findings for term to preterm pigs.

### Intestinal Responses

The higher relative intestinal mass for preterm pigs fed the two LP milk replacers compared with the HP-Control (HP = 38.9 ± 5.4 vs. LP = 41.5 ± 5.7; *P* = 0.03) coincides with the increased rate of body weight gain (21). A novel finding of clinical importance is the growth responses of the intestine to protein content and supplemental leucine were evident for the smaller (<1,000 g) but not the larger (>1,000 g) pigs. It is uncertain if supplementing the LP milk replacer with HMB would elicit a similar dichotomy in growth responses for smaller and larger pigs. It is possible the enhanced small intestine development of preterm pigs in response to colostrum (33, 34) may be even more pronounced for those considered as SGA. Although prematurity is considered to reduce the stimulation of protein synthesis induced by feeding (35) and possibly by leucine and other growth promoters, the present findings highlight the need to consider body weight.

The higher lactase activity of the preterm pigs compared with maltase and maltodextrinase (present study) corresponds with the evolutionary milk diet, the low tolerance of suckling pigs for non-lactose carbohydrates, and the increased incidence of necrotizing enterocolitis when preterm pigs are fed formula with maltodextrin (27). The responses of the three brush border membrane carbohydrases to the LP and HP milk replacers and supplemental leucine were again evident for the smaller but not the larger preterm pigs. The nearly 50% increase in total intestinal carbohydrase activities of the smaller pigs fed the LP-Leu and HP-Control milk replacers should allow the SGA pigs to tolerate dietary loads of lactose and alternative carbohydrates that are larger relative to body weight.

The lack of differences in rates of glucose uptake and total intestinal capacities to transport glucose suggest a limited ability to use increased protein or growth promoters to increase glucose uptake. An estimate of the amount of glucose that can be absorbed by the intestines of the preterm pigs (15.5 g/kg-day) is not in great excess of consumption based on the composition and volume of milk replacer fed (6.25 g/kg-day) but would greatly exceed the amount of glucose that would be consumed as a fetus. Although the responses to different dietary loads of glucose were not examined, the ability of pigs to adaptively regulate glucose uptake may not develop until after birth corresponding with when dietary carbohydrate intake would start to fluctuate (36).

The consequences of the different forskolin induced chloride secretion by the proximal intestines of preterm pigs fed the LP and HP milk replacers is uncertain but may indicate a reduced risk of diarrhea (37).

### Responses of Other Organs

Organ growth is asymmetric in fetal humans (38, 39), as it is for fetal pigs of varying ages (40) and for preterm pigs at the same gestational age but of different body weights (Present study; Table 4). As a result, the proportion of preterm pig body mass allocated to specific organs varies with body size at the stage of post-conception age studied. While some organs grow in direct proportion with body weight (hearts), others grow slower (lung, gastrointestinal tract, and especially the brain), and some faster (liver, kidneys, pancreas and spleen). Based on data from human fetuses (38, 39) the proportion of body weight allocated to different organs will vary at different stages of gestation. The present study indicates growth of the kidneys after preterm birth is responsive to protein intake but the relationship with nephrogenesis and renal functions later in life is unknown.

Typically, about 10% of newborn pigs weigh <800 g at term due to intrauterine growth restriction (41, 42). We considered the 19 preterm pigs that were <700 g at 105 days of gestation (~15%) as SGA. These pigs were <1,000 g at necropsy and those fed the two LP milk replacers grew at a lower rate (percent gain per day) when compared to larger pigs fed the same formulas (*P* < 0.05). The differences in growth rate for the small and large preterm pigs were not evident for the HP formulas. The term SGA and AGA pigs provide insights into organ size if growth continues until term. The similar relative organ weights, with the exception of the brain, for the SGA and AGA term pigs suggest growth remains matched with the gain of body weight. The SGA pigs had smaller intestines as previously reported (43), but not when normalized to body weight. The different relative organ weights of the preterm and term pigs, despite small differences in post-conception ages (112–113 and 115 days, respectively) suggest preterm birth and the standards of care used for this study altered the normal trajectories of development for some organs. Notably, the term pigs had suckled, but the rapid changes in intestinal weight and functions in response to colostrum (44) did not result in relative larger intestines and livers compared with the preterm pigs. This may reflect a prolonged tropic response to the 6 or 7 days the preterm pigs were fed.

Neurodevelopment is of particular interest for infants born preterm, and particularly those that are SGA. During normal gestation the human brain remains a consistent proportion of body weight [slope = 0.96; from ref (38, 39)], which led to the model of brain sparing proposed by Barker that has been verified for human neonates (45). However, SGA infants have smaller, less developed brains (46) and are at greater risk of delayed neurodevelopment (47), as do preterm infants that suffer EUGR (2). Although brain weight of fetal pigs increases faster than body weight between days 60 and 110 of gestation [slope = 1.38; from ref (40)], brain weight for the preterm pigs that weighed >1,000 g at necropsy was independent of body weight and averaged 30.7 g across this range of body weights. Like SGA infants, the brains of pigs that were <700 g at delivery (<1,000 at necropsy) were smaller and correspond with the smaller and less developed brains of newborn term SGA pigs [(48); present study]. Apparently, the potential for brain sparing is limited. Fetuses below a critical weight (pigs or humans) are unable to reallocate sufficient nutrients from other tissues to allow for normal brain development. Furthermore, proliferation and migration of cerebellar granule cells and maturation of Purkinje cells in the pig cerebellum may be especially sensitive to prematurity and growth restriction (49, 50). Although the present study does not indicate leucine and HMB can mitigate the reduced growth of the brain and cerebellum, a phosphatidylserine source of docosahexanoic acid has improved cerebellum development after preterm birth (51, 52).

### Perspectives

The preterm pig is a relevant large animal model for understanding the combined influences of how being born early and small influences growth and developmental responses to nutrition support strategies and the potential for lifelong consequences (53). There is a critical need to identify ingredients that will promote normal growth and development of the gut and other organs after preterm birth (54, 55).

## Data Availability Statement

The raw data supporting the conclusions of this article will be made available by the authors, without undue reservation.

## Ethics Statement

The animal study was reviewed and approved by University of Memphis Institutional Animal Care and Use Committee.

## Author Contributions

RB: designed, performed experiments, collected and analyzed samples, experiments, prepared manuscript. TY: performed experiments, collected and analyzed samples, analyzed and interpreted data. AA, VC, and II: collected and analyzed samples and contributed to manuscript preparation. KB: designed, performed experiments, supervised animal care and use, interpreted data. All authors contributed to the article and approved the submitted version.

## Conflict of Interest

The authors declare that the research was conducted in the absence of any commercial or financial relationships that could be construed as a potential conflict of interest.
